# Speed tuning properties of mirror symmetry detection mechanisms

**DOI:** 10.1038/s41598-019-39064-x

**Published:** 2019-03-05

**Authors:** Rebecca J. Sharman, Elena Gheorghiu

**Affiliations:** University of Stirling, Department of Psychology, Stirling, FK9 4LA Scotland United Kingdom

## Abstract

The human visual system is often tasked with extracting image properties such as symmetry from rapidly moving objects and scenes. The extent to which motion speed and symmetry processing mechanisms interact is not known. Here we examine speed-tuning properties of symmetry detection mechanisms using dynamic dot-patterns containing varying amounts of position and local motion-direction symmetry. We measured symmetry detection thresholds for stimuli in which symmetric and noise elements either drifted with different relative speeds, were relocated at different relative temporal frequencies or were static. We also measured percentage correct responses under two stimulus conditions: a segregated condition in which symmetric and noise elements drifted at different speeds, and a non-segregated condition in which the symmetric elements drifted at two different speeds in equal proportions, as did the noise elements. We found that performance (i) improved gradually with increasing the difference in relative speed between symmetric and noise elements, but was invariant across relative temporal frequencies/lifetime duration differences between symmetric and noise elements, (ii) was higher in the segregated compared to non-segregated conditions, and in the moving compared to the static conditions. We conclude that symmetry detection mechanisms are broadly tuned to speed, with speed-selective symmetry channels combining their outputs by probability summation.

## Introduction

Mirror symmetry (henceforth ‘symmetry’) is an image property where one half of a stimulus reflects the other about an axis. Symmetry is a salient feature for the human visual system and can be found throughout natural and man-made structures. Humans detect symmetry quickly and automatically^1–4^. Psychophysical, brain imaging and computational modelling studies have shown that symmetry perception plays a role in figure-ground segregation^5–7^, object recognition^8–10^, visual search^11^ and amodal completion^12,13^, thus, providing insight into the ways in which the human visual system solves fundamental perceptual problems. Symmetry elicits a distinct pattern of brain activity involving a wide network of extra-striate visual areas including V3a, V4, V7 and LOC^14^. Although recent psychophysical studies have examined luminance polarity and colour^15–20^, binocular^21–23^, and temporal^24–26^ properties of symmetry perception, it remains to be established how symmetry detection mechanisms interact with those involved in motion-speed processing. It is known that the visual system groups local stimulus elements according to their speed (and direction) of movement, a process that is constrained by two competing processes: a segmentation process which detects changes in speed across space and parses the image into regions of independent speeds, and an integration process which smooths out local speed changes and links elements of different local speed to the same moving object or region^27–29^. On the other hand, stimulus layout has been also found to affect the efficacy of that segmentation, i.e., parsing an image into separate regions improves speed discrimination, an indicator of more effective segmentation^30^. If spatial layout affects segmentation by speed, then there may be differences in how the visual system processes symmetrical and non-symmetrical stimuli. Therefore, we examine whether symmetry detection mechanisms are tuned or selective to the speed of pattern elements. That is, does the visual system have symmetry mechanisms that are *gated* by speed? For example, a mechanism that groups all elements of one speed and derives a symmetry-signal for that particular speed, another that groups all elements of a different speed and derives another speed symmetry signal, and so on.

Recent studies have shown that symmetry detection in dynamic stimuli is subject to a cumulative process in which weak symmetry signals are integrated by spatiotemporal filters over ~120 ms, thus, resulting in increased overall signal strength^24–26^. This suggests that element locations can be combined over time, and thus, increasing the number of symmetrical element locations improves performance in symmetry detection tasks^24,26^. By definition drifting pattern elements have different successive element locations over time and the faster the elements drift the more locations there will be. Thus, it might be the case that the temporal rate at which elements change locations, rather than the speed of elements, affects symmetry detection. Neurophysiological evidence has shown that tuning for temporal frequency and tuning for speed are separate neuronal properties^31,32^, however, we are not aware of studies that found temporal frequency tuning for specific visual processes e.g., depth, form, texture, or symmetry perception.

Selectivity to speed has been found for a number of visual processes, such as contour detection in noise^33^, shape processing in context^34,35^, motion in depth^36^. Detection of spatial contours drifting at one speed is enhanced by speed differences between background and contour elements, a finding which has been explained by the Gestalt law of ‘shared common fate’ (i.e., segmentation by speed and/or motion direction) rather than by the law of ‘good continuity’ (i.e., contour integration)^33^. Contextual modulation effects on the shape of contours as evidenced by the surround suppression phenomenon has been also found to be selective for speed and motion direction^34^. These selectivities are in keeping with one aim of vision being to segregate contours that define objects from those that form textured surfaces^34,35^. Although existing evidence suggests that speed can be used to perform this kind of image segmentation, it remains unclear whether this process is accomplished by specialised mechanisms or a general, all-purpose speed-based image-segmentation mechanism^37^. With regard to symmetry, recent studies have shown that although symmetrical local *motion-direction* does not contribute to symmetry detection, limiting the lifetime of pattern elements does improve performance^24,26^. However, to our knowledge no study has examined whether symmetry detection mechanisms are *tuned to the speed* of symmetrical motions.

There is some neurophysiological evidence for speed tuning in the cortex, which also supports the possibility of symmetry detection mechanisms tuned for speed. Neurophysiological studies have shown that one quarter of V1 complex cells are sensitive to speed, with their preferred speed being invariant of spatial frequency^38^. Higher visual areas, such as area MT, inherit these responses which are subsequently used to compute selectivity for speed^38–40^. fMRI adaptation studies have found evidence for speed encoding in V1, V2, V3a, V3b and V4, although this tuning may be inherited from V5^39^. Some of these visual areas are also involved in symmetry perception (V3a, V4), thus, making it plausible that some symmetry detection mechanisms might be tuned for speed.

In this communication, we will examine whether symmetry detection mechanisms are tuned for the speed of symmetrical motion. To examine the contribution of elements speed to symmetry perception, none of the stimulus conditions will contain coherent *global* motion which could be used to segregate symmetry and noise by motion direction. In our stimuli, it will only be possible to group or segregate elements by speed. Thus, matched pairs will move symmetrically with either one speed or two different speeds, but there will be no coherent pattern of global motion. We will measure symmetry detection thresholds using a two-interval forced-choice task (2IFC). In each trial, a target stimulus containing variable amounts of symmetry will be presented in one interval and a foil stimulus containing random-positioned dots will be presented in the other. Participants will indicate by a key press which interval contains the symmetric stimulus.

**Figure 1:**
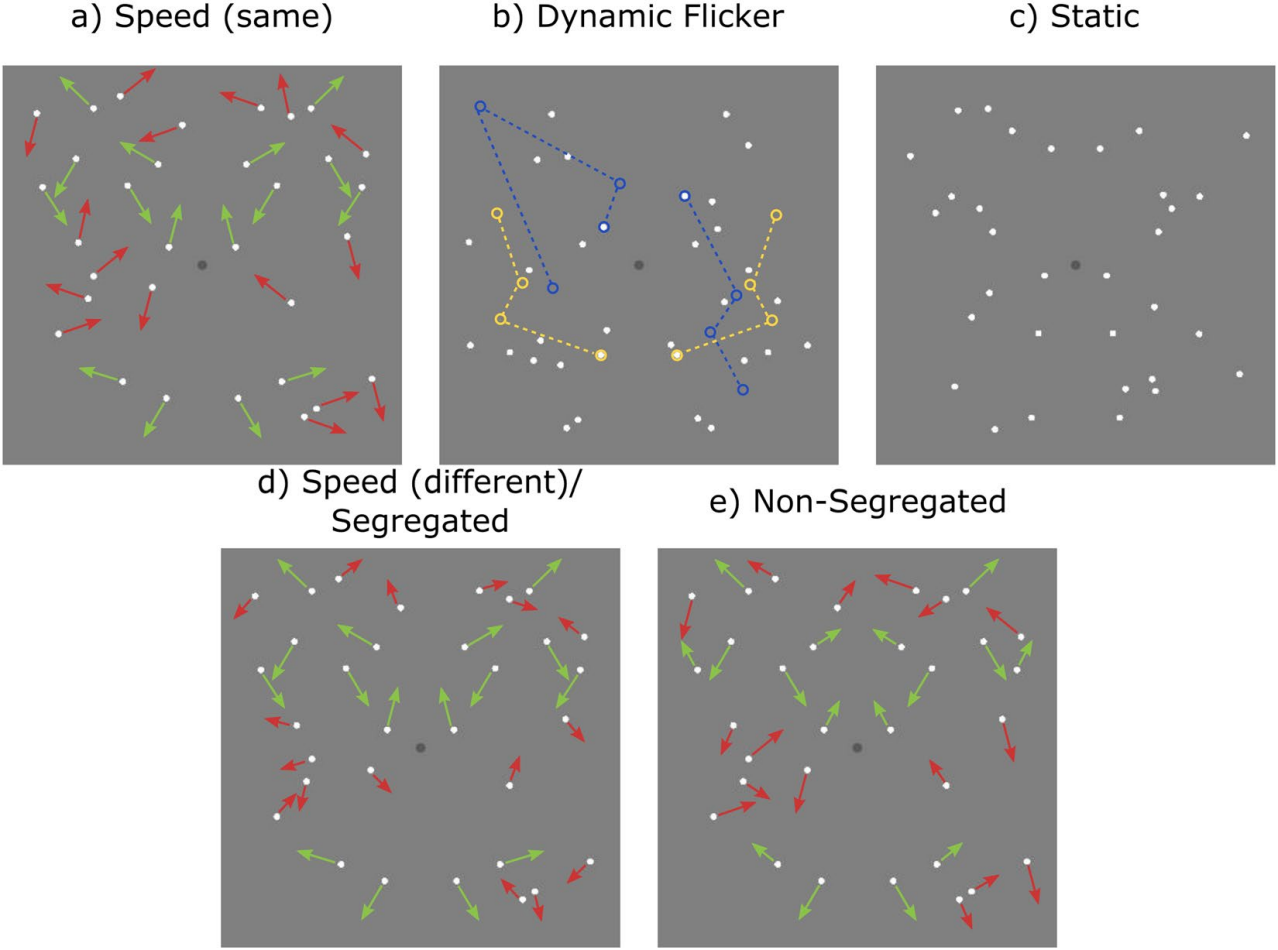
A schematic representation of stimuli used in Experiment 1 (**a**–**c**) and Experiment 2 (**d**,**e**). The length of the green and red arrows indicates the speed of symmetrical and noise dots, respectively. (**a**) Speed condition in which symmetric and noise dots drift with either the same (**a**) or different (**d**) speeds. (**b**) Dynamic flicker condition in which elements had a limited lifetime and were relocated to multiple, random successive locations, without any coherent motion. Blue and yellow circles connected by dashed lines represent hypothetical, successive locations of a single pair of dots when relocating over time to different locations (dashed lines are for illustration only, there was no motion trajectory present in the stimulus). (**c**) Static condition in which a single, static pattern was presented for the entire stimulus duration. (**d**,**e**) In Experiment 2, all stimuli contain 50% position symmetry and there were two conditions: (**d**) a segregated condition in which symmetric dots drift in symmetric, but random directions at one speed and noise dots drift at another speed, and (**e**) non-segregated condition, in which half of the symmetric pairs and noise dots moved at one speed and the other half of symmetric and noise dots moved at another.

First, we will examine whether symmetry detection mechanisms are sensitive to the relative speed differences between the symmetric and noise pattern elements. To test this, we measure symmetry detection thresholds for patterns in which symmetrical pairs drift at one speed and noise elements drift at a different speed (see Fig. 1a and Movie S1). We vary the relative *speed difference* between the symmetric and noise elements and predict that symmetry detection thresholds will improve with increasing speed difference. However, if we find an improvement then this might be also due to the temporal dynamics rather than speed of the elements. This is because in order to keep the maximum distance travelled by each dot constant across the different speed conditions (1.18 deg), the dots will take different amounts of time to travel that distance for each speed condition (352.94 for 3.33 deg/s, 176.47 for 6.67 deg/s and 117.65 ms for 10 deg/s). Hence, the lifetime duration of pattern elements will vary as a function of speed. In order to dissociate between temporal dynamics and motion speed, we will also measure symmetry detection thresholds for patterns in which elements have limited lifetimes, but there is no local motion, i.e., dynamic flicker condition (Fig. 1b and Movie S2). Therefore, if we find any differences between the speed and dynamic flicker conditions then these will be attributable to the speed of the pattern elements. We also predict that symmetry detection thresholds will be lower for the speed conditions compared to the dynamic flicker conditions due to the increase in the number of element locations. Furthermore, all speed and dynamic flicker conditions will be compared to a static condition in which a single pattern is displayed for the whole duration of the stimulus presentation (Fig. 1c). The static condition provides a baseline to which the other dynamic conditions can be compared. In accordance with our previous findings we predict higher thresholds for the static condition compared to all dynamic (i.e. speed and dynamic flicker) conditions^24,26^. In sum, this experiment will indicate whether symmetry detection mechanisms are sensitive to the local speed differences of pattern elements and whether these can be used to segment symmetric and noise patterns.

In a second experiment, we will examine whether symmetry detection mechanisms are selective for speed. Put another way, whether the visual system has positional grouping mechanisms that are gated by speed. To test for this, we will compare performance (i.e. the percentage of correct responses) under two conditions: a ‘segregated’ condition in which the symmetrical dots drift at one speed and noise dots drift at another speed (Fig. 1d) and a ‘non-segregated’ condition in which the symmetrical dot-pairs were drifting with two different speeds in equal proportions, as were the noise or random dots (Fig. 1e). To allow for direct comparison between segregated and non-segregated conditions, the patterns always contained 50% position-symmetric elements while the remaining elements were randomly positioned, and the speed of the symmetric and noise patterns was varied between 3.33, 6.67 and 10 deg/s. Furthermore, we have designed our segregated condition in such a way to prevent the speed of the symmetric pattern from ‘popping out’ and capturing the observers’ attention. In our segregated condition the speed of the symmetric pattern was randomly selected from the two possible stimulus speeds, thus, preventing observers from knowing which speed contained the symmetry signal. If there are speed-selective symmetry channels, we expect performance to be better in the segregated than non-segregated condition. This is because in the segregated condition the symmetry signal is carried by just one speed, but in the non-segregated condition it is carried by two speeds. On the assumption that if speed-selective symmetry channels exist the independent signals from them would be combined by probability summation, then we predict better performance in the segregated than non-segregated condition. In the segregated condition, the symmetry signals would be in a single, speed-selective symmetry channel, producing a strong symmetry signal in that channel and no signal in the other speed-selective symmetry channel. On the other hand, in the non-segregated condition, the two speed-selective symmetry channels would only receive half-strength signals and thus, will be weakly activated, due to the presence of noise dots drifting at the same speeds. Based on the experimental data obtained for the segregated condition, we will derive predictions of the percentage of correct responses in the non-segregated condition, based on the assumption of probability summation of independent speed-selective symmetry channels within the framework of signal detection theory. If the predicted data are a good fit for the experimental data for the non-segregated condition, then this will provide evidence for the existence of independent, speed-selective symmetry channels that are combined by probability summation.

## Results

### Experiment 1: Are symmetry detection mechanisms tuned for speed?

**Figure 2:**
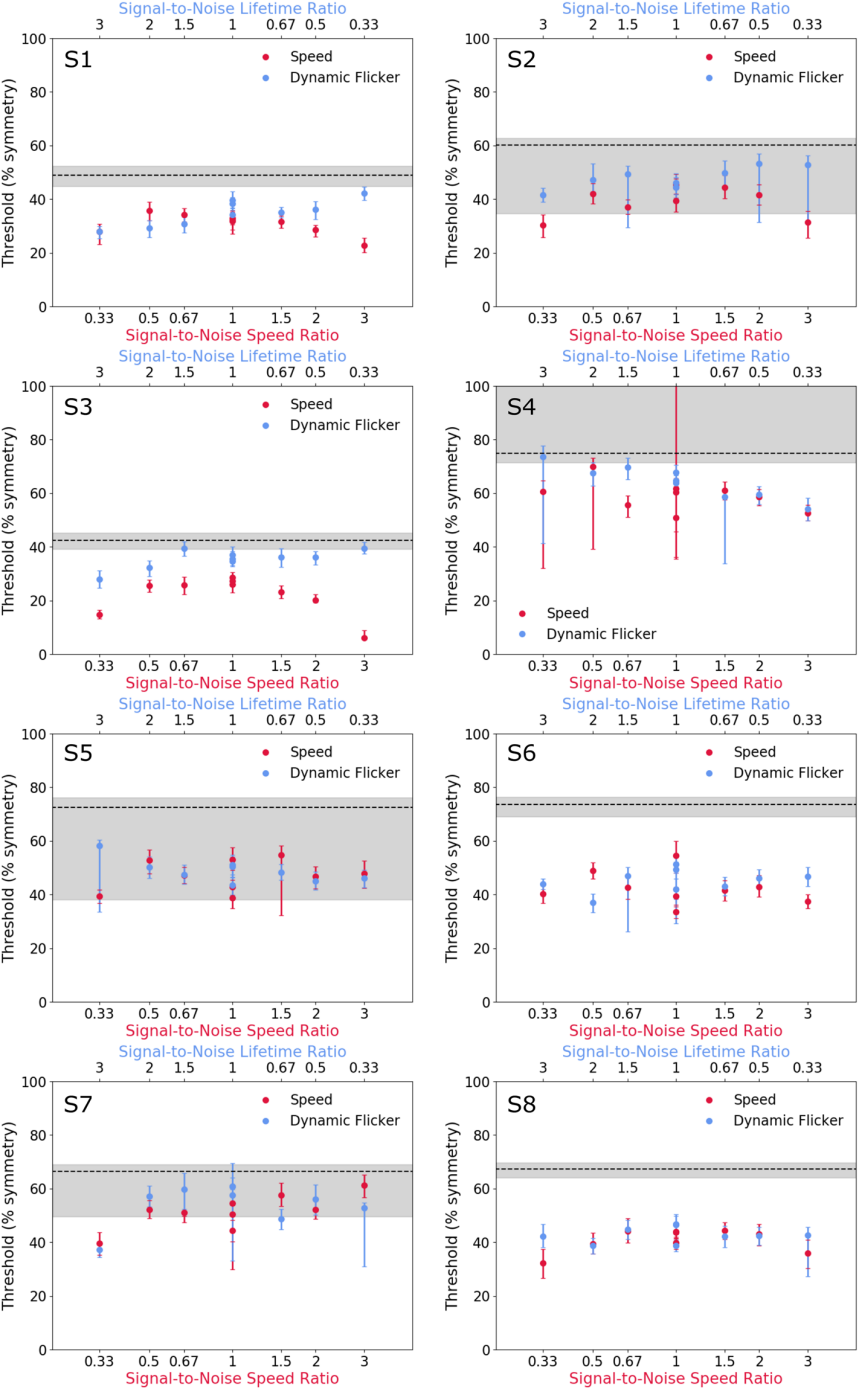
Symmetry detection thresholds for the speed (red symbols) condition as a function of signal-to-noise speed ratio (lower axis), for each observer. The thresholds for the dynamic flicker (blue symbols) condition are shown as a function of signal-to-noise lifetime ratio (upper axis). The static condition is indicated by the dashed line. Error bars are standard error (SE) generated from 1000 bootstrap resamples.

**Figure 3:**
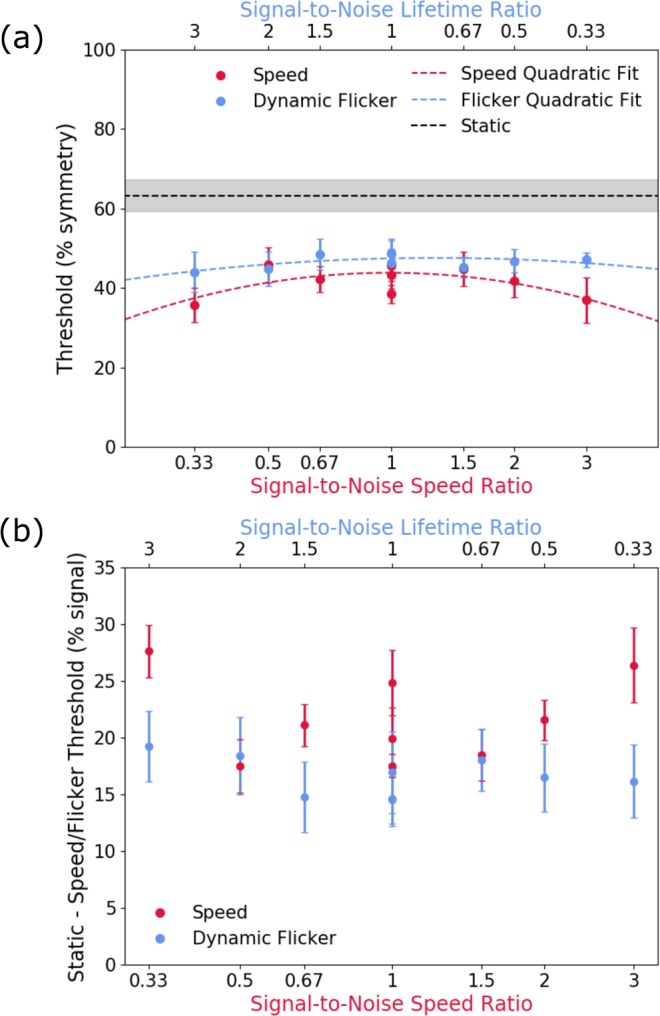
Average across-observers (**a**) symmetry detection thresholds and (**b**) difference thresholds calculated as static – speed (or flicker) thresholds. The speed conditions (red symbols) are shown as a function of signal-to-noise speed ratio (lower axis), while the flicker condition is plotted as a function of signal-to-noise lifetime ratio (upper axis). The static condition is represented by the black dashed line in top panel (**a**), while in bottom panel b (difference from static) it corresponds to zero. The red and blue dashed lines in the top panel (**a**) represents quadratic functions fit to the speed thresholds (red) and dynamic flicker thresholds (blue). The signal-to-noise ratios, which were calculated as speed (or lifetime) of symmetrical elements divided by speed (or lifetime) of noise elements, are plotted in reverse for the dynamic flicker condition as a larger ratio represents a longer lifetime duration and therefore, a slower rate of change, whereas for the speed condition a larger ratio represents a longer distance travelled and therefore a faster rate of movement. Hence, in order to compare shorter lifetimes with faster speeds, the ratios of signal-to-noise must be plotted in reverse order. Error bars and grey area are ±1 standard error of the mean (SEM).

Figure 2 shows symmetry detection thresholds for the speed (red symbols) condition as a function of signal-to-noise speed ratio (lower axis), for each observer. The thresholds for the dynamic flicker (blue symbols) condition are shown as a function of signal-to-noise lifetime ratio (upper axis) and the static condition is indicated by the dashed line. The average across-participants thresholds are shown in Fig. 3a. Figure 2 indicates lower thresholds for the two dynamic conditions compared to the static condition in all participants, with thresholds for the speed condition being slightly more reduced than those for the dynamic flicker condition.

To compare the reduction in absolute thresholds between observers, we normalised the results to the static condition for each observer, by computing a difference threshold between the static and dynamic conditions. Figure 3b shows the averaged across-observers difference in symmetry detection thresholds (% symmetry signal) between the static and speed (red symbols) conditions as a function of the symmetry signal-to-noise speed ratio. The difference threshold between the static and dynamic flicker (blue symbols) conditions are also shown as a function of the signal-to-noise ratio of element lifetime duration (upper axis). Higher difference values in Fig. 3b represent improved performance as the thresholds for the dynamic conditions were subtracted from the static condition. The results show that for the speed condition, thresholds were slightly higher when symmetric and noise elements drifted with the same speed (i.e. signal-to-noise speed ratio of 1) and decreased gradually as the speed difference between signal and noise increased (red symbols). However, for dynamic flicker conditions, thresholds were comparable across the different signal-to-noise lifetime durations (blue symbols).

A two-way repeated-measures analysis of variance (ANOVA) with factors, stimulus condition (motion speed vs. dynamic flicker) and signal-to-noise ratio (0.33, 0.5, 0.67, 1, 1.5, 2, 3) was carried out on the symmetry detection threshold data. The three conditions where the signal and noise dots had the same speed or the same lifetime (i.e. signal-to-noise ratios of 1) were considered as separate data points. The analysis revealed that the thresholds in the speed condition were significantly lower than those in the dynamic flicker condition (F(1,7) = 13.206, p = 0.008, ƞ^2^ = 0.654). There was also a significant effect of signal-to-noise ratio (F(8,56) = 4.359, p = 0.001, ƞ^2^ = 0.384) and a marginally significant interaction between the two factors (F(8,56) = 2.139, p = 0.047, ƞ^2^ = 0.234). Bonferroni corrected post-hoc analysis showed that this interaction was driven by a significant difference between the condition in which both signal and noise dots moved at 3.33 deg/s and the condition in which symmetrical dots moved at 3.33 deg/s and noise dots moved at 10 deg/s (t(7) = 5.767, p = 0.025).

We also ran a one-way repeated-measures ANOVA to further examine how signal-to-noise speed ratio (or lifetime duration ratio) affected thresholds in the speed (or dynamic flicker) condition. The analysis revealed a significant effect of signal-to-noise speed ratio in the speed condition (F(8,56) = 5.475, p = 0.001, ƞ^2^ = 0.439), and no significant effect of signal-to-noise lifetime ratio in the dynamic flicker condition (F(8,56) = 1.010, p = 0.439, ƞ^2^ = 0.126). In addition, Bonferroni corrected t-tests showed that all thresholds obtained in both the speed and dynamic flicker conditions were significantly lower than those obtained with the static stimuli (p < 0.05).

These results indicate that symmetry detection mechanisms are broadly tuned for the signal-to-noise speed ratio, suggesting that larger speed differences can aid segmentation of symmetry from noise signals. To examine this broad speed-tuning, we fitted a quadratic function to the speed data (red line in Fig. 3a), with coefficients a = −5.321, b = −0.133 and c = 43.819. We also fitted a quadratic function to the dynamic flicker data (blue line in Fig. 3a), with coefficients a = −1.808, b = 0.906, c = 47.455. These two quadratic curves were significantly different from each other (F(3,12) = 6.892, p = 0.006).

However, no lifetime duration/temporal frequency tuning is present in the dynamic flicker conditions, thus demonstrating that the observed tuning is driven by speed-sensitive mechanisms and not by temporal frequency/lifetime duration. These results demonstrate that symmetry detection mechanisms are sensitive to the relative speed differences between symmetric and noise pattern elements.

### Experiment 2: Are symmetry detection mechanisms selective for speed?

**Figure 4:**
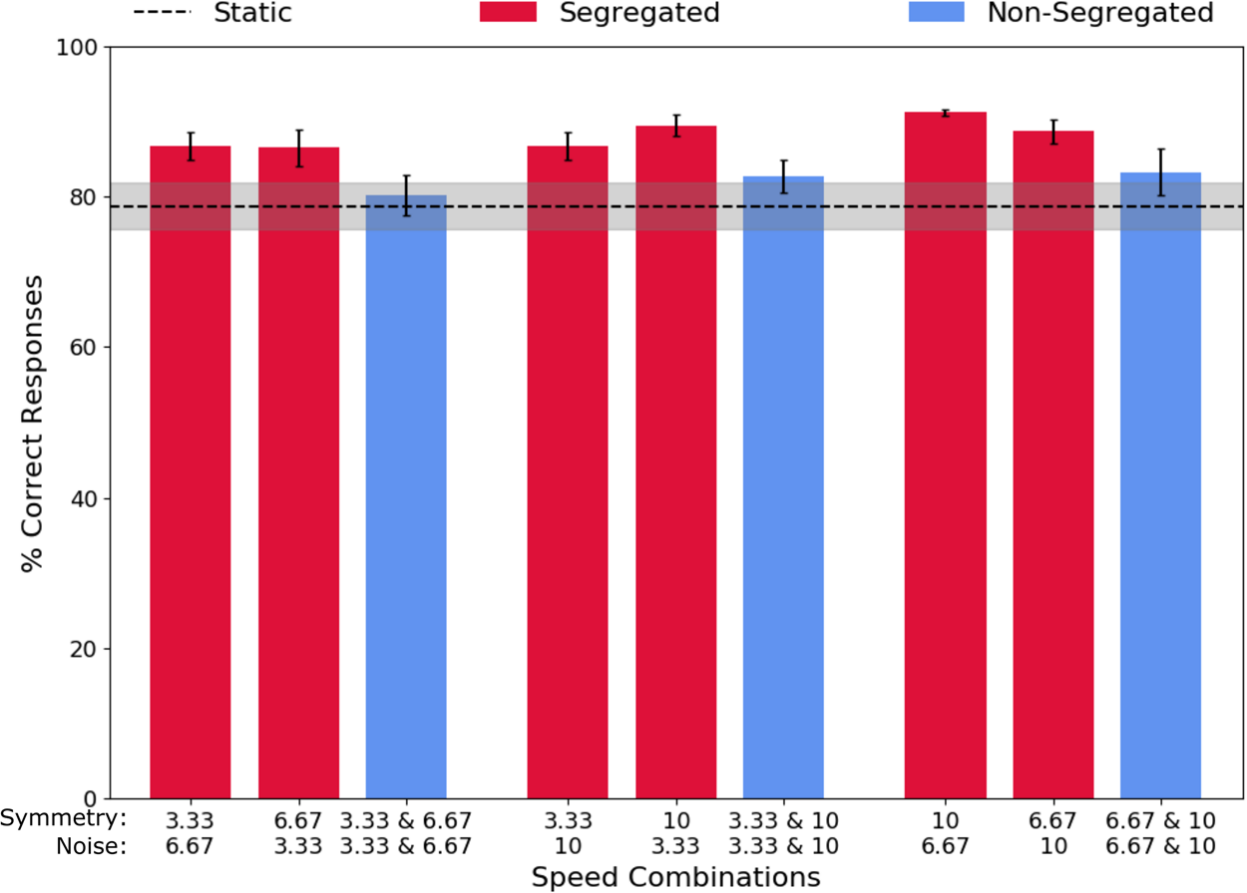
Average across-observers of the percentage of correct responses in the segregated (red bars), non-segregated (blue bars), and static (dotted line) conditions. Higher values indicate better performance. Error bars and grey band are ±1 SEM.

Figure 4 shows that average across-observers percentage correct responses for the segregated (red circles) and non-segregated (blue circles) conditions, as a function of the speed combinations of the symmetry signal and noise. The percentage correct responses for the static condition are indicated by the black dashed line. The results show better performance in the segregated compared to the non-segregated condition and static condition.

A one-way repeated measures ANOVA on the logit transformed data showed no significant differences between any of the segregated conditions (F(5,15) = 0.7, p = 0.632, ƞ^2^ = 0.189). Thus, we averaged the two segregated conditions corresponding to the component speeds in the non-segregated condition (e.g., the segregated conditions with 3.33 deg/s symmetry speed and 6.67 deg/s noise speed was averaged with the condition with 6.67 deg/s symmetry speed and 3.33 deg/s noise speed, and so on).

A two-way repeated measures ANOVA with factors stimulus condition (segregated vs. non-segregated) and stimulus speed combinations (0.33 and 0.67, 0.33 and 10, 6.67 and 10) was carried out on the logit transformed percentage correct data. There was a significant difference between the segregated and non-segregated speed conditions (F(1,3) = 12.811, p = 0.037, ƞ^2^ = 0.810). However, there was no significant effect of stimulus speed combination (F(2,6) = 1.257, p = 0.350, ƞ^2^ = 0.295), and no significant interaction (F(2,6) = 0.187, p = 0.834, ƞ^2^ = 0.059. Overall, these results indicate that symmetry detection mechanisms are selective or tuned to speed.

### Probability summation model

In order to test whether the relationship between the segregated and non-segregated data supports the presence of independent speed-selective symmetry channels, we used a signal detection theory analysis of probability summation described by Kingdom and colleagues^41^ and implemented in the Palamedes toolbox^42^. We used the segregated data in which all symmetry signals were carried by one speed, to derive the predicted percentage correct data for the non-segregated condition in which the symmetry signals were carried by two different speeds in equal proportion. Assuming probability summation of independent speed-selective symmetry channels, we make the following assumptions: (a) if speed-symmetry channels exist then they will contribute independent signals to detection by probability summation, (b) the strength of the symmetry signal in each speed-symmetry channel is proportional to the percentage of symmetric dots drifting at that particular speed, and (c) the observers monitor the information in all speed-sensitive symmetry channels for which there are speeds in the stimulus. Based on these assumptions, performance in the segregated condition results from the probability summation of one, full strength (i.e., 16 symmetric pairs drifting at speed S_1_) and one, zero strength (i.e., 0 symmetric pairs drifting at speed S_2_) signal, and in the non-segregated condition from the probability summation of two, half-strength (8 symmetric pairs drifting at speed S_1_ and 8 symmetric pairs drifting at speed S_2_).

Within the Palamedes toolbox, the equation for the probability summation of equal intensity stimuli is implemented by two routines: PAL_SDT_PS_PCtoSL, which converts percentage correct to stimulus intensity level and PAL_SDT_PS_SLtoPC, which does the reverse. The first routine has six arguments: percentage correct (PC), a scaling factor *g* which converts signal intensity to the d-prime (d′) measure, τ the exponent of the transducer function (i.e. the function that relates perceived to physical symmetry), *M* the number of alternatives in the forced-choice task, *Q* the number of monitored channels, in this case two as there are two speeds, and *n* the number of channels that are activated (i.e. that contain a stimulus). The output of this routine is a value of intensity or stimulus level (SL) which corresponds to the probability-summed strength of the symmetry signal. Given our segregated condition, we input the measured PC, set *g* and τ to unity, M to 2 as we used a 2IFC task, the number of monitored channels Q to two as there are two speeds in each stimulus and n to 1 as in the segregated condition only one speed carried the symmetry signal. To determine the predicted PC for the non-segregated condition we used the second routine PAL_SDT_PS_SL to PC. The SL value from the first routine was then divided by the number of speeds and input to the second routine and n was set to the number of speeds. All other input parameters were the same as those used before.

**Figure 5:**
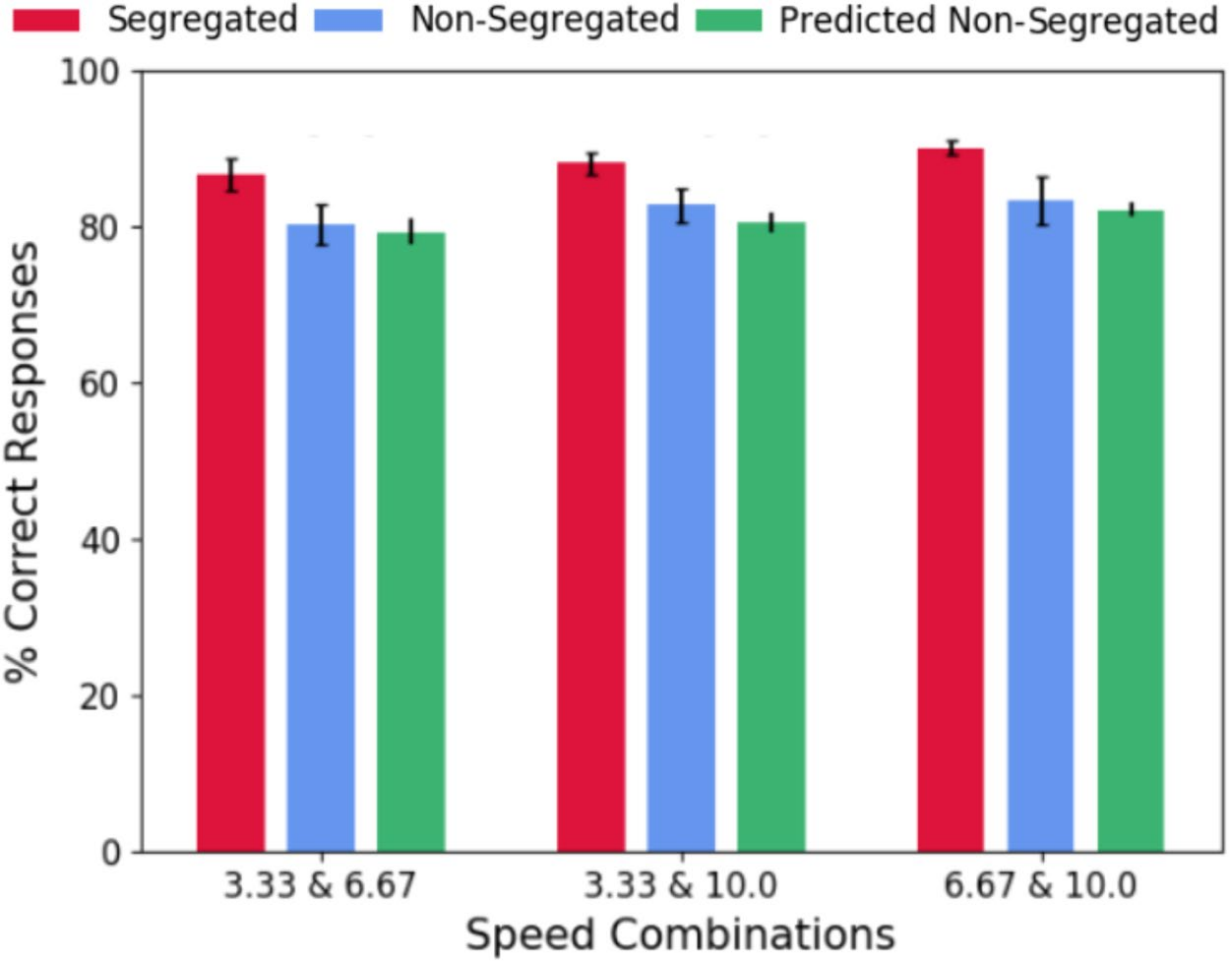
Probability summation of independent speed-symmetry channels model predictions. Average across-observers predicted non-segregated data (green bars) are shown together with the experimental data for the non-segregated (blue bars), and segregated (red bars) conditions. Error bars are ±1 SEM.

Figure 5 shows the average across-observers model predictions obtained for the percentage correct in the non-segregated condition (green symbols), together with the experimental data for the segregated (red symbols) and non-segregated (blue symbols) conditions. The predicted percentage correct based on the probability summation model is slightly lower than the measured percentage correct for all conditions.

A two-way repeated-measures ANOVA was performed on the logit transformed data with factors type of non-segregated data (experimental vs. predicted) and speed combinations (0.33 and 0.67 vs. 0.33 and 10.01 vs. 6.67 and 10.01 deg/s). The analysis revealed that there was no significant difference between the model predictions and the experimental data (F(1,3) = 0.631, p = 0.485, ƞ^2^ = 0.174), therefore indicating that independent speed-selective symmetry channels combine their signals by probability summation. In addition, there was no significant effect of speed combinations (F(2,6) = 1.181, p = 0.369, ƞ^2^ = 0.282), and no significant interaction between the type of non-segregated data and the different speed combinations (F(2,6) = 0.141, p = 0.872, ƞ^2^ = 0.045).

## Discussion

We examined whether symmetry mechanisms are tuned or selective to the speed of pattern elements. We found that symmetry detection thresholds (i) decreased gradually as the speed differences between symmetry signal and noise elements increased, and (ii) remained of comparable magnitude across all dynamic flicker (no coherent motion) conditions. Our results show that symmetry detection thresholds are broadly tuned to the speed of symmetric/matched pairs and this selectivity is not explained by changes in element lifetime/temporal frequency of the pattern. Furthermore, we demonstrated that broadly-tuned, independent speed-selective symmetry channels are combined by probability summation.

Locher and Wagemans^43^ have suggested that the ‘virtual lines’ that exist between all neighbouring elements in a pattern^44^ may contribute to the symmetry signal, by serving as primitives in the construction of the primal sketch^45^. In our speed condition, the trajectory of the dots may have created or reinforced symmetrical ‘virtual lines’. These ‘virtual lines’ were not present in the dynamic flicker condition, as despite the relocation of the dots being symmetrical, it was not possible to track the relocation of particular dot pairs. Increased speed could also increase the amount of ‘virtual lines’ present in the stimuli. However, as dots always travelled the same distance before being relocated, the length of the ‘virtual lines’ would remain the same. If ‘virtual lines’ do contribute to the symmetry signal this could underpin the speed tuning observed in the current study.

The results suggest that symmetry mechanisms can use the outputs of image segmentation by motion speed to facilitate symmetry detection. Our results are consistent with other findings on the role of motion speed in perceptual organisation^37^. For example, Hess and Ledgeway^37^ reported that contour detection in noise improves as the difference between signal and noise element speed increases. In a control experiment, they demonstrated that this sensitivity to speed differences was also present when dots were grouped by spatial location i.e. clustered dots moved at a different speed to the background. Thus, these authors suggested that the visual system has a ‘general-purpose’ speed-based image-segmentation mechanism rather than one which is specific to contour detection.

Other studies examined the effect of a surround texture on the perceived shape of a contour using shape adaptation and found that shape after-effects induced in single contour tests are reduced when the adaptor contour is surrounded by a texture made of similarly shaped contours, a phenomenon termed texture-surround suppression of contour shape^34^. By varying the relative speed differences between the central contour and surround texture adaptor, these studies found that this surround-suppression is also selective for speed^34^, thus indicating that speed differences can facilitate the segregation of contours that are parts of objects from those that are parts of textures.

The visual system segments scenes by grouping local stimulus elements by their speed or movement direction. Stimulus layout affects this process, when elements are spatially grouped together segmentation by speed is improved^30^. We have shown that when symmetric elements are segregated by speed, symmetry detection performance improves. Grouping by speed facilitates symmetry detection, potentially by improving segmentation and making it easier for the visual system to disregard randomly positioned noise elements. This is in contrast to findings showing that grouping by symmetric motion-direction does not improve performance^24^. This contrast is not necessarily surprising as there is some evidence suggesting that speed and motion direction processing are underpinned by temporally and spatially distinct mechanisms^46,47^.

The percentage of correct responses observed in the non-segregated condition are consistent with what we would expect if there were independent speed-selective symmetry channels combined by probability summation. However, it remains to be determined whether this speed-selectivity of symmetry detection mechanisms is inherited from earlier speed selective visual areas (e.g., V1, V2), or whether it is specific to the visual areas involved in symmetry perception (e.g., V3a, V4). In other words, does the selectivity for speed shown by symmetry detection mechanisms originate from a generalised speed-based image segmentation mechanism located in an earlier visual area? Alternatively, evidence from neuropsychological studies suggests that the intraparietal sulcus (IPS) is necessary for visual processing of global regularity in Glass patterns^48^. This implies that dorsal areas are necessary for global pattern coding in intermediate ventral regions^48^. Support for this, comes also from neurophysiological studies showing that non-directional V4 neurons can acquire motion-direction selectivity after motion adaptation, as a result of feedback from higher areas such as MT and MST^49^. Therefore, dorsal areas might be responsible for speed selectivity in intermediate visual areas (e.g. V3, V4) involved in symmetry perception found in this study. This is also in line with other psychophysical studies suggesting that global motion-direction selectivity of surround suppression of shape phenomena might be caused by feedback from MT/MST^34^.

There are two different categories of computational models for detecting symmetry: models based on complex grouping rules from which symmetry is subsequently extracted^50^ and models using early spatial mechanisms e.g., oriented filters combined using an AND-like operation^51,52^ and/or in a filter-rectify-filter model^53,54^. If we assume that symmetry is computed using a filter-rectify-filter model^54^, similar to texture processing^55^, which requires integration over large spatial areas, then how could speed selectivity be inherited from V1 and V2, when neurons in these areas have small receptive fields which do not integrate across large spatial regions? In order for outputs from V1 and V2 with small receptive fields to contribute to symmetry perception, the visual system may group the elements by speed and then subsequently decide whether a given speed pattern is symmetric. Hence, it is likely that selectivity to speed of symmetry detection mechanisms occurs directly in the mid-to-higher visual areas V3 and V4, given that these visual areas are involved in both symmetry and speed encoding^14,39^.

What visual features have symmetry detection mechanisms been found to be selective for? Previous studies have shown that although symmetry detection mechanisms *are sensitive* to colour and luminance-polarity correlations across the symmetry axis, they are *not selective* or gated for these features^15,17^. The detectability of symmetry was also found to be unaffected by noise when the symmetric and noise patterns were located in different depth planes^22^, but it deteriorated considerably when the two symmetric halves were located in different depth planes^23^ suggesting that symmetry detection is sensitive to 3D spatial correlation across the symmetry axis. However, it has not yet been determined whether symmetry is selective for disparity. Here we showed that symmetry detection mechanisms are selective for speed which supports the important role of speed in figure-ground segregation, as well as symmetry detection.

## Method

### Participants

Eight observers participated in the first experiment (the first author and seven observers who were naïve with regard to the experimental aims) and four observers (including the first author) participated in the second experiment. All observers had normal or corrected-to-normal vision. Observers gave their written informed consent prior to participating in the study and were treated in accordance with the Declaration of Helsinki (2008, Version 6). All procedures were approved by the Psychology Ethics Committee, University of Stirling, UK.

### Stimulus – Generation and Display

Stimuli were presented on a gamma-corrected 20-in ViewSonic, Professional Series PF817 cathode ray tube (CRT) monitor (ViewSonic, Brea, CA, USA) with spatial resolution 1024 × 768 and a refresh rate of 85 Hz. A ViSaGe MKII stimulus generator (Cambridge Research Systems, Cambridge, UK) in Bits# mode was used to control contrast. All stimuli were presented in the centre of the monitor on a mid-grey background with average luminance of 47.2 cd/m^2^. Viewing distance was 57 cm. All stimuli were generated and all data were collected using PsychoPy^56^.

Stimuli were presented in a square window 13.74° in width and were comprised of 32 circular full contrast white dots of 0.24° diameter resulting in a dot density of 0.17 dots/deg^2^. Symmetrical dots were randomly positioned on the left side of the square area and then mirrored about the vertical axis onto the right side. Noise dots were randomly positioned, with equal numbers appearing in each stimulus half.

Stimuli were dynamic dot patterns consisting of symmetrical dot-pairs drifting in symmetrical directions, but different pairs had different randomly allocated directions of motion. The symmetric dot-pairs and noise dots drifted with either the same or different speed (see Fig. 1a for a schematic illustration and Movie S1 for a dynamic version). The elements in all dynamic stimuli had a limited-lifetime, such that after the maximum lifetime duration was reached the element ‘died’ and was relocated to a new random location. Starting ‘ages’ were randomly allocated such that different dot pairs reached their maximum lifetime and ‘died’ at different times. Each pair of symmetrical dots was relocated simultaneously in order to maintain the same level of symmetry throughout presentation, but with different pairs relocating asynchronously. In conditions with element motion, the distance travelled by each pattern element before it was relocated was kept constant (1.18°), as a result lifetime duration varied as a function of element speed. Specifically, element speeds of 3.33, 6.67 and 10 deg/s have lifetime durations of 352.94, 176.47 and 117.65 ms respectively.

In Experiment 1, there were three stimulus conditions: (1) ‘speed’ conditions in which symmetrical dot-pairs drifted in symmetrical directions, with different pairs having randomly allocated motion directions. The motion direction of the noise dots was randomly allocated for those in the left half of the stimulus, those directions were then mirrored and randomly allocated to the elements in the right half of the stimulus, to ensure the same distribution of speeds in both halves of the stimulus (Fig. 1a and Movie S1 for a dynamic version). We varied the speed of the symmetric and noise dots relative to each other. We used three speed values: 3.33, 6.67 and 10 deg/s, therefore resulting in nine symmetry and noise speed combinations. (2) ‘dynamic flicker’ conditions in which signal and noise dots had no local or global motion, but their lifetime duration was limited (Fig. 1b and Movie S2). We used three different element lifetime durations: 117.65, 176.47 and 352.94 ms, which correspond to temporal frequencies of 8.5, 5.67 and 2.83 Hz, respectively. These values correspond to the lifetime durations of the three different speeds in the speed conditions. (3) static stimulus condition in which a single pattern was presented for the entire trial (Fig. 1c). We measured and compared symmetry detection thresholds for the three stimulus conditions.

In Experiment 2, we examined whether symmetry detection mechanisms are gated by speed. In all conditions, the patterns contained 32 elements which were divided equally into 16 dots drifting at one speed S_1_ and 16 dots drifting at another speed S_2_. All patterns contained 50% position symmetry and dot pairs moved in symmetrical directions, but different pairs had different randomly allocated directions of motion. There were two symmetric stimulus conditions: (1) a ‘segregated’ condition in which the symmetric pairs drifted at one speed and noise dots drifted at a different speed (Fig. 1d and Movie S3), and (2) a ‘non-segregated’ condition in which the symmetric dots drifted at two different speeds in equal proportions and so did the noise dots (Fig. 1e and Movie S4). Note that the amount of positional symmetry is identical for the segregated and non-segregated conditions (compare Fig. 1d,e in which the stimuli contain 50% position symmetry). The foil or without–symmetry comparison stimuli contained the same distribution of motion-directions and speeds as the with symmetry stimuli but with all elements being randomly positioned.

### Procedure

In Experiment 1, a two-interval forced-choice (2IFC) procedure was employed to measure symmetry detection thresholds. In each trial, a target stimulus containing variable amounts of symmetry between 0 and 100% symmetry was presented in one interval and a foil stimulus consisting of randomly positioned dots was presented in the other. The distribution of speeds and motion-directions was the same in each interval. For example, if the target interval contained 6 symmetrical dots moving at speed S_1_ and 26 dots moving at speed S_2_, the foil interval would contain 6 dots moving at speed S_1_ and 26 dots moving at speed S_2_, all of which would be randomly positioned. All dots moved in randomly allocated directions. Each stimulus was presented for 400 ms with an inter-stimulus interval (ISI) of 400 ms. The presentation order of the target and foil intervals was randomised from trial to trial. The participants’ task was to indicate, by a key press, which interval contained the symmetrical stimulus.

In each trial, we measured the minimum number of symmetric dots required for the participant to perceive the pattern as symmetrical (i.e. symmetry detection threshold). Thresholds were measured using a one-up, three-down staircase procedure. The staircases controlled the number of symmetrical dots in the target stimulus. In each run, two staircases were interleaved, one starting with 100% symmetry in the target and the other starting with 0% symmetry. The conditions themselves were not interleaved. The staircases were designed to converge at the 79.37% threshold and were terminated after 75 trials. Participants were allowed as many practice runs as required to become familiar with the task. Each participant collected a minimum of ten staircases for each condition (750 trials). For each participant and each experimental condition, correct responses were averaged for each amount of stimulus symmetry and a logistic function was fit to these data. Thresholds were then estimated from these fits as the stimulus intensity level at which the observer performed at 80% probability of responding correctly.

In Experiment 2, we used a 2IFC procedure and measured the percentage of correct responses given by each participant. The procedure was the same as for Experiment 1, except that in all trials the target patterns contained 50% symmetrical dots. Segregated conditions (i.e. signal and noise dots drifting at different speeds) comprised of the same component speeds were randomly interleaved (i.e. the condition where the speed of symmetric elements was 3.33 deg/s and noise speed was 10 deg/s was interleaved with the condition where symmetrical elements’ speed was 10 deg/s and noise speed was 3.33 deg/s). This meant that participants could not predict or learn the speed of the symmetric dots from successive trials. Each participant collected a minimum of 100 trials for each speed combination condition. For each participant, correct responses were averaged to calculate the percentage of correct responses for each stimulus condition.

## Supplementary material

VideoS1

VideoS2

VideoS3

VideoS4

## References

[1] Bertamini M, Makin ADJ (2014). Brain Activity in Response to Visual Symmetry. Symmetry-Basel.

[2] Allen G (1879). The origin of the sense of symmetry. Mind.

[3] Tyler, C. W. *Human symmetry perception and its computational analysis*. (Psychology Press, 2003).

[4] Bertamini M, Silvanto J, Norcia AM, Makin ADJ, Wagemans J (2018). The neural basis of visual symmetry and its role in mid- and high-level visual processing. Annals of the New York Academy of Sciences.

[5] Driver J, Baylis GC, Rafal RD (1992). Preserved figure ground segregation and symmetry perception in visual neglect. Nature.

[6] Machilsen B, Pauwels M, Wagemans J (2009). The role of vertical mirror symmetry in visual shape detection. J. Vis..

[7] Makin ADJ, Rampone G, Wright A, Martinovic J, Bertamini M (2014). Visual symmetry in objects and gaps. J. Vis..

[8] Pashler H (1990). Coordinate frame for symmetry detection and object recognition. J. Exp. Psychol. Hum. Percept. Perform..

[9] Vetter T, Poggio T (1994). Symmetrical 3D objects are an easy case for 2D object recognition. Spat. Vis..

[10] Vetter T, Poggio T, Bulthoff HH (1994). The importance of symmetry and virtual views in 3-dimensional object recognition. Curr. Biol..

[11] Wolfe JM, Friedmanhill SR (1992). On the role of symmetry in visual-search. Psychol. Sci..

[12] Saiki J (2000). Occlusion, symmetry, and object-based attention: Comment on Behrmann, Zemel, and Meter (1998). J. Exp. Psychol.-Hum. Percept. Perform..

[13] van Lier RJ, Vanderhelm PA, Leeuwenberg ELJ (1995). Competing global and local completions in visual occlusion. J. Exp. Psychol.-Hum. Percept. Perform..

[14] Sasaki Y, Vanduffel W, Knutsen T, Tyler C, Tootell R (2005). Symmetry activates extrastriate visual cortex in human and nonhuman primates. Proc. Natl. Acad. Sci. USA.

[15] Gheorghiu E, Kingdom FA, Remkes A, Li HC, Rainville S (2016). The role of color and attention-to-color in mirror-symmetry perception. Sci. Rep..

[16] Morales D, Pashler H (1999). No role for colour in symmetry perception. Nature.

[17] Wright D, Mitchell C, Dering BR, Gheorghiu E (2018). Luminance-polarity distribution across the symmetry axis affects the electrophysiological response to symmetry. Neuroimage.

[18] Wu CC, Chen CC (2014). The Symmetry Detection Mechanisms are Color Selective. Sci. Rep..

[19] Wu CC, Chen CC (2017). The Integration of Color-Selective Mechanisms in Symmetry Detection. Sci. Rep..

[20] Martinovic J, Jennings BJ, Makin ADJ, Bertamini M, Angelescu I (2018). Symmetry perception for patterns defined by color and luminance. J. Vis..

[21] Erkelens CJ, van Ee R (2007). Monocular symmetry in binocular vision. J. Vis..

[22] Ishiguchi A, Yakushijin R (1999). Does symmetry structure facilitate the depth separation between stereoscopically overlapped dot planes?. Percept. Psychophys..

[23] Treder MS, van der Helm PA (2007). Symmetry versus repetition in cyclopean vision: A microgenetic analysis. Vision Res..

[24] Sharman RJ, Gheorghiu E (2017). The role of motion and number of element locations in mirror symmetry perception. Sci. Rep..

[25] Sharman RJ, Gregersen S, Gheorghiu E (2018). Temporal dynamics of mirror-symmetry perception. J. Vis..

[26] Sharman, R. J. & Gheorghiu, E. Spatiotemporal and luminance contrast properties of symmetry perception. *Symmetry-Basel* **10** (2018).

[27] Mestre DR, Masson GS, Stone LS (2001). Spatial scale of motion segmentation from speed cues. Vision Res..

[28] Masson GS, Mestre DR, Stone LS (1999). Speed tuning of motion segmentation and discrimination. Vision Res..

[29] Braddick O (1993). Segmentation versus integration in visual-motion processing. Trends in Neurosciences.

[30] Martin A, Barraza JF, Colombo EM (2009). The effect of spatial layout on motion segmentation. Vision Res..

[31] Perrone JA, Thiele A (2002). A model of speed tuning in MT neurons. Vision Res..

[32] Foster KH, Gaska JP, Nagler M, Pollen DA (1985). Spatial and temporal frequency-selectivity of neurons in visual cortical areas V1 and V2 of the macaque monkey. J. Physiol.-London.

[33] Hess RF, Ledgeway T (2003). The detection of direction-defined and speed-defined spatial contours: one mechanism or two?. Vision Res..

[34] Gheorghiu E, Kingdom FAA (2017). Dynamics of contextual modulation of perceived shape in human vision. Sci. Rep..

[35] Gheorghiu E, Kingdom FAA, Petkov N (2014). Contextual modulation as de-texturizer. Vision Res..

[36] Wardle SG, Alais D (2013). Evidence for speed sensitivity to motion in depth from binocular cues. J. Vis..

[37] Ledgeway T, Hess RF (2002). Rules for combining the outputs of local motion detectors to define simple contours. Vision Res..

[38] Priebe NJ, Lisberger SG, Movshon JA (2006). Tuning for spatiotemporal frequency and speed in directionally selective neurons of macaque striate cortex. J. Neurosci..

[39] Lingnau A, Ashida H, Wall MB, Smith AT (2009). Speed encoding in human visual cortex revealed by fMRI adaptation. J. Vis..

[40] Priebe NJ, Cassanello CR, Lisberger SG (2003). The neural representation of speed in macaque area MT/V5. J. Neurosci..

[41] Kingdom FAA, Baldwin AS, Schmidtmann G (2015). Modeling probability and additive summation for detection across multiple mechanisms under the assumptions of signal detection theory. J. Vis..

[42] Prins, N. & Kingdom, F. A. A. *Palamedes: Matlab routines for analysing psychophysical data*, http://www.palamedestoolbox.org (2009).

[43] Locher PJ, Wagemans J (1993). Effects of element type and spatial grouping on symmetry detection. Perception.

[44] Stevens KA (1978). Computation of locally parallel structure. Biological Cybernetics.

[45] Marr, D. *A computational investigation into the human representation and processing of visual information*. (W H Freeman, 1982).

[46] Kubota T, Kaneoke Y, Maruyama K, Watanabe K, Kakigi R (2004). Temporal structure of the apparent motion perception: a magnetoencephalographic study. Neurosci. Res..

[47] Mercier M, Schwartz S, Michel CM, Blanke O (2009). Motion direction tuning in human visual cortex. Eur. J. Neurosci..

[48] Lestou V, Lam JML, Humphreys K, Kourtzi Z, Humphreys GW (2014). A Dorsal Visual Route Necessary for Global Form Perception: Evidence from Neuropsychological fMRI. Journal of Cognitive Neuroscience.

[49] Tolias AS, Keliris GA, Smirnakis SM, Logothetis NK (2005). Neurons in macaque area V4 acquire directional tuning after adaptation to motion stimuli. Nature Neuroscience.

[50] Scognamillo R, Rhodes G, Morrone C, Burr D (2003). A feature-based model of symmetry detection. Proc. R. Soc. B-Biol. Sci..

[51] Cohen EH, Zaidi Q (2013). Symmetry in context: Salience of mirror symmetry in natural patterns. J. Vis..

[52] Rainville, S.J. & Kingdom, F.A.A. The functional role of oriented spatial filters in the perception of mirror symmetry - psychophysics and modellings. J.Vis. 40, 9, 2621-2644 (2000) 10.1016/s0042-6989(00)00110-3 10958913

[53] Poirier F, Wilson HR (2010). A biologically plausible model of human shape symmetry perception. J. Vis..

[54] Dakin SC, Watt RJ (1994). Detection of bilateral symmetry using spatial filters. Spat. Vis..

[55] Kingdom FAA, Prins N, Hayes A (2003). Mechanism independence for texture-modulation detection is consistent with a filter-rectify-filter mechanism. Visual Neurosci..

[56] Peirce JW (2007). PsychoPy - Psychophysics software in Python. J. Neurosci. Methods.

