# Supplementary figures and images for: Speed tuning properties of mirror symmetry detection mechanisms
Source: Sci Rep. 2019 Mar 5;9:3431. doi: 10.1038/s41598-019-39064-x (PMC6400945; doi:10.1038/s41598-019-39064-x)

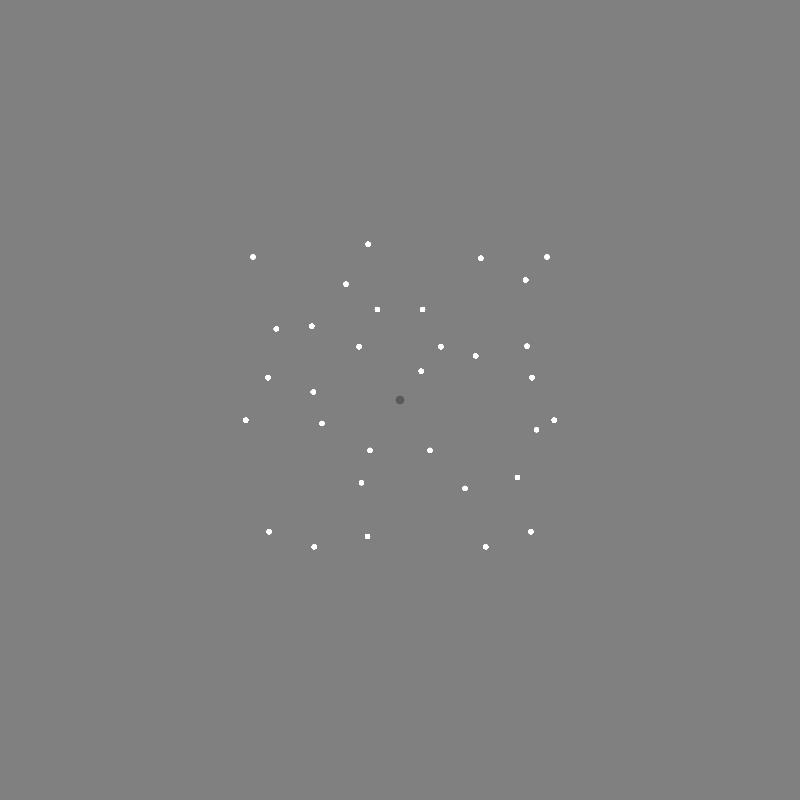

Supplement: Supplementary file 1 — VideoS1 [file 41598_2019_39064_MOESM1_ESM.gif]

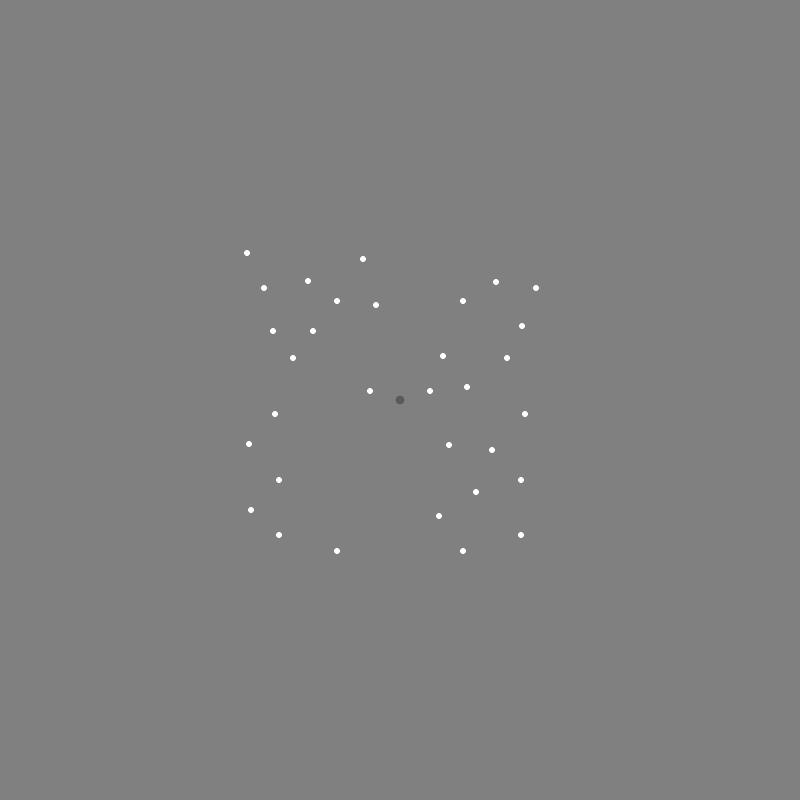

Supplement: Supplementary file 2 — VideoS2 [file 41598_2019_39064_MOESM2_ESM.gif]

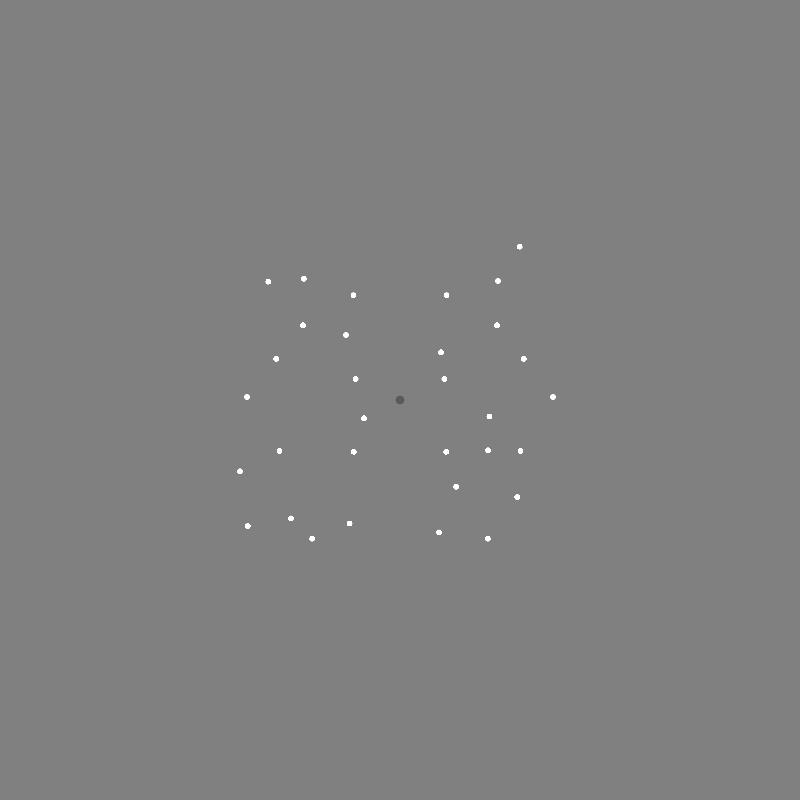

Supplement: Supplementary file 3 — VideoS3 [file 41598_2019_39064_MOESM3_ESM.gif]
